# Critical Review on the Significance of Olive Phytochemicals in Plant Physiology and Human Health

**DOI:** 10.3390/molecules22111986

**Published:** 2017-11-16

**Authors:** Irene Gouvinhas, Nelson Machado, Carla Sobreira, Raúl Domínguez-Perles, Sónia Gomes, Eduardo Rosa, Ana I. R. N. A. Barros

**Affiliations:** 1 Centre for the Research and Technology of Agro-Environmental and Biological Sciences, CITAB, University of Trás-os-Montes and Alto Douro, UTAD, Quinta de Prados, 5000-801 Vila Real, Portugal; nmachado@utad.pt (N.M.); csobreira@utad.pt (C.S.); rdperles@utad.pt (R.D.-P.); erosa@utad.pt (E.R.); abarros@utad.pt (A.I.R.N.A.B.); 2 University of Trás-os-Montes and Alto Douro, 5000-801 Vila Real, Portugal; 3 BioISI–Biosystems & Integrative Sciences Institute, Faculty of Sciences, University of Lisboa, Campo Grande, 1649-004 Lisboa, Portugal; sgomes@utad.pt

**Keywords:** *Olea europaea* L., mediterranean diet, phenolic compounds, molecular structure, biological activity

## Abstract

Olive oil displays remarkable organoleptic and nutritional features, which turn it into a foodstuff appreciated by consumers, and a basic component of the Mediterranean diet. Indeed, the noticed benefits of including olive oil in the diet have been assigned to the presence of diverse bioactive compounds with different molecular structures. These compounds confer a wide range of biological properties to this food matrix, including the prevention of distinct human diseases as well as the modulation of their severity. The most relevant bioactive compounds present in olive oil correspond to benzoic and cinnamic acids, phenolic alcohols and secoiridoids, and also flavonoids. Over the last decades, several studies, devoted to gaining a further insight into the relative contribution of the separate groups and individual compounds for their biological activities, have been conducted, providing relevant information on structure–activity relationships. Therefore, this paper critically reviews the health benefits evidenced by distinct phenolic compounds found in olive oils, thus contributing to clarify the relationship between their chemical structures and biological functions, further supporting their interest as essential ingredients of wholesome foods.

## 1. Introduction

The olive tree (*Olea europaea* L.) is the only representative of the *Oleaceae* family producing edible fruits, this culture displaying the capacity to survive in poor soils and under drought and semi-drought growing conditions. Besides the productive characteristics of olive crops and their relevance for the agro-food industry, olive tree fruits and their by-products represent valuable sources of nutrients and non-nutrients, responsible for the nutritional and sensory properties, as well as for the biological activities and health benefits attributed to edible olives and olive oils [1].

The major purpose of olive tree culture is the production and sale of olive oil, mainly virgin olive oil, which is among the most important components found in the Mediterranean diet. This oil is obtained from healthy and intact olives exclusively by mechanical processing (crushing, malaxation, and centrifugation), thus maintaining their phytochemical and nutritional composition. Regarding olive oil quality, the International Olive Council [2] has defined different grades according to chemical composition and degree of acidity [2], the best brand corresponding to Extra Virgin Olive Oil (EVOO). For the achievement of this classification, an olive oil must contain less than 0.8 g of free acids per 100.0 g, expressed as oleic acid [3], besides presenting no noticeable organoleptic defects. The sensory characteristics of this product, which are highly appreciated by consumers, are due to a complex mixture of volatile compounds, including aldehydes, alcohols, ketones, esters, and hydrocarbons, which have been lately identified and quantified [4]. Alteration of the normal organoleptic characteristics can occur during storage, as a consequence of the peroxidation of fatty acids responsible for rancidity, which ultimately results in the formation of volatile compounds with negative sensory features. This process leads to a quality decrease and deleterious effects on human health, which is due to the presence of free radicals [5].

Besides the appealing organoleptic characteristics, the increasingly growing demand for olive oil is associated with the well-established correlation of its consumption with the promotion of human health [6]. In this sense, several differences between diverse olive oils have been described regarding their chemical composition. For instance, unlike refined oils, virgin olive oil does not contain chemicals resulting from the solvents used in the refining process, which ultimately contributes to preserve the original properties and constituents of this product [7]. In addition to the relevance of the sensory characteristics of olive oil, its consumption has been stressed as a source of valuable bioactive phytochemicals during the last decades. Therefore, the composition of the final marketable product on bioactive compounds, contributes to the maintenance of the normal physiological status and the prevention of distinct pathological conditions related to oxidative stress, such as cancer, cardiovascular diseases, metabolic disorders, and inflammation [4], the antioxidant power being highlighted as the most relevant function of this fraction. Actually, this functional property, due to the bioactive compounds, allows the modulation of oxidative reactions responsible for diverse pathophysiological situations.

## 2. Olive Fruits

The olive tree (*Olea europaea* L.) produces olive fruits, which are rarely used in their natural raw form because of the requirement of specific processing to avoid the high fruit bitterness. Also, in addition to the olives directed for consumption, most of the harvested crop is used by the industry to produce olive oils.

Olives are fruits of relatively small size, with about 1.0 to 4.0 cm long and 0.6 cm to 2.0 cm in diameter, presenting an elliptical shape. The weight of an olive varies from 3 to 20 g [8]. Furthermore, this fruit presents three main structures: endocarp (seed), mesocarp (pulp), and exocarp (skin). In ripe olives, the pulp represents 84–90% of the total fruit weight, while the seed accounts from 13 to 23%, and epidermis 2–3% [9]. Concerning the basic chemical composition, olives contain components with high nutritional value including: lipids, sugars, and proteins, besides water and some minerals [9].

Apart from the nutrients provided by these fruits, over the last years, the presence of interesting non-nutrient compounds with positive effects on the sensory characteristics of olives, as well as on human health, has been described. This non-nutrient fraction, mainly represented by phenolic compounds, accounts for up to 3% of the olives fresh weight [10]. The major classes of phenolic compounds present in olives are phenolic acids, phenolic alcohols, secoiridoids, and flavonoids [11], which are present in almost all tissues of the fruit, although their concentrations vary greatly between the distinct parts [12]. Regarding individual compounds, the hydroxytyrosol derivatives, oleuropein, verbascoside, and ligstroside, represent some of the most important ones [13].

Hydroxytyrosol, belonging to the class of the phenolic alcohols, alongside tyrosol, are among the most abundant compounds in olive fruits, their concentration increasing throughout the ripening process, as a result of the hydrolysis of oleuropein, the major secoiridoid constituent in the unripe olive fruit. This glucoside (oleuropein) is responsible for the bitter taste of green olives [14,15]. Furthermore, in addition to the role as a precursor of simpler compounds (hydroxytyrosol, ligstroside, and oleuropein aglycone, among others), oleuropein is associated with some beneficial effects on human health. According to Omar [16], oleuropein presents antioxidant, anti-hypertensive, and anti-inflammatory activity, and protects the body against cardiovascular diseases.

Flavonoids are also important compounds in olives, being represented by luteolin-7-glucoside, luteolin-5-glucoside, apigenin-7-glucoside, apigenin-7-rutinoside, and quercetin-3-rutinoside. Their concentrations increase during the maturation process [17].

Since phenolic compounds are part of the plant response to stress, their concentration in olives, and therefore in olive oils, has been demonstrated to be the result of complex interactions between diverse factors, including agronomical features (cultivar and geographical origin, agricultural practices, fruit ripening, and irrigation regime) and industrial processes (extraction and storage procedures) [18,19,20,21,22].

### 2.1. Agronomic Features of Olives and Olive Oils: Effect on Composition and Quality

The potential uses, within the agro-food industry, of distinct olive cultivars, are directly associated with the phytochemical content and basic chemical composition of olive fruits, and consequently, of the oils obtained. In this regard, the composition of these foodstuffs (olives and olive oils) regarding the phenolic compounds, results from the complex interaction between several factors, namely, genetic factors (cultivar) and agro-climatic conditions (abiotic and biotic stress, such as cultivation/agronomic practices, and pathogen outbreaks). Despite the effect of these factors on the phenolic composition, they also influence olive oils, regarding the composition in fatty acids and volatile compounds, as well as concerning quality parameters (such as free acidity, peroxide value, and K_270_ and K_232_), and the sensory properties.

Besides genetic and agronomic factors, several authors have reported changes in the phenolic content and profile as a consequence of the processing methods applied for oil obtaining, including pressing, malaxation, centrifugation, and filtration. These processing methods and the storage conditions have been evaluated regarding their influence on the polyphenolic composition [18,20,23,24,25,26,27,28].

### 2.2. Cultivar

The relevance of genetic factors concerning the synthesis of phenolic compounds in olive fruits is mirrored by the large differences existing between olive tree cultivars. However, this fact not only influences the phytochemical composition of olives, but also exerts a huge effect on the phenolic content in olive oils. With respect to the genetic diversity, there are distinct cultivars of *O. europaea* L., producing olive fruits with a considerable diversity in size, shape, oil-content and flavour, as well as regarding biochemical characteristics. This diversity allows to obtain olive oils with specific composition.

Concerning the physical characteristics, the shapes range from almost round to oval or elongated with pointed ends, and the size varies from small to very big (3–20 g). Besides these physical properties, over the last years, several authors have reported significant differences with respect to the phenolic composition of virgin olive oils (VOO) from Portuguese, Turkish, and Spanish cultivars [29,30,31]. In this connection, the phenolic compositions of diverse Portuguese cultivars were studied by Vinha et al. [22], showing that the polyphenolic composition varies considerably between cultivars at matching ripening stages [32]. This fact turns the phenolic compounds present in VOO into valuable markers to establish geographical origins and/or cultivars.

### 2.3. Maturation Stage

As previously mentioned, the composition of olives undergoes several changes throughout the ripening process, and thus, the maturation stage of olives constitutes a relevant issue, in which respects to the chemical composition of olives, and their corresponding oil. In this concern, the olive fruits ripening index (RI) is one of the most important traits related to the quality of olive fruits and oils. The relevance of this factor has been attributed to the changes occurring at different maturation stages, which comprise physiological, biochemical, metabolic, and enzymatic features [33].

Olive ripening lasts several months and varies according to the growing area, cultivar, water availability, temperature, and agricultural practices. Thus, the development of olive fruits is characterized by a rapid growth in the initial stages, followed by a subsequent slower stage and an accelerated growth step [34]. In general, advanced maturation results in an increase of phenolic content to a maximum level at the "half pigmentation" stage, decreasing according to the progress of the season [35].

**Figure 1:**
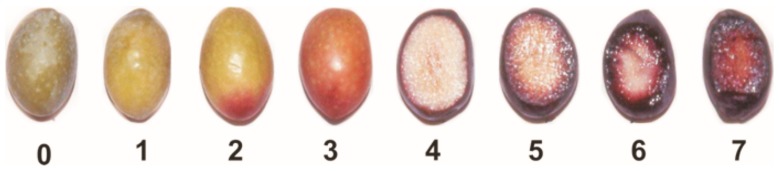
Ripening index: 0, skin colour deep green; **1**, skin colour yellow green; **2**, Skin colour green with reddish spots on <half fruit surface; **3**, skin colour with >half fruit surface turning reddish or purple; **4**, skin colour black with white flesh; **5**, skin colour black with <half flesh turning purple; **6**, skin colour black with not all the flesh purple to the stone; **7**, skin colour black with all the flesh purple to the stone.

The skin and pulp colour is one of the most relevant attributes for assessing ripening (Figure 1). However, since distinct maturation timings have been assigned as optimal harvesting stage for different cultivars, an RI was developed by Uceda and Hermoso [36], as a valuable tool for the producers to categorize olives. For the determination of the RI, the drupes were evaluated according to skin and pulp colour. Values range from 0 (100% intense green skin) to 7 (100% purple flesh and black skin).

### 2.4. Irrigation Regime

In the last few years, some studies have been undertaken regarding the effects of irrigation in olive orchards [37,38,39,40]. The olive tree is a drought-tolerant species suitable to be grown in semi-arid areas with limited water resources. Regarding water shortage, in modern intensive and super-intensive olive groves, irrigation is a critical factor due to the relationship of its management with, not only the reduction of the production costs, but also the increase of yield and quality parameters [41]. The major effects linked to an adequate irrigation in olive orchards are the increases in: fruit size, number of fruits per tree, fruit production, and oil content of drupes [42,43].

Concerning the effect of irrigation on olive oil quality, the studies available so far did not show any variation regarding peroxide value, free acidity, and UV absorption parameters at 232 and 270 nm [44,45,46], whilst polyphenolic contents were the most affected by irrigation. The latter, are not only affected in terms of total composition, but also concerning their profiles. Furthermore, irrigation did not affect the separate individual phenolics in the same way, although the content of phenolic compounds has been shown to generally decrease with irrigation [39,47,48]. However, seemingly contradictory data, reported by other authors, allowed to conclude that, given the physiology of olive trees, water availability is considered a plant stress, which affects the secondary metabolism, increasing the synthesis of phenolic compounds [49,50]. Actually, these data obtained from distinct authors seem to be compatible, since, if in one hand the metabolism of secondary metabolites is increased by irrigation, on the other hand, their apparent concentration is lower due to the increased size of the fruits, resulting in a final balance that could lead to significant differences in the phenolics’ concentration in either ways, to lower or higher values.

### 2.5. Geographical Origin

To date, several studies have classified distinct olive oils with respect to their geographical origin, by taking advantage of the influence of the edaphoclimatic conditions on the compositional and sensorial characteristics of olives and olive oils. In this sense, the phenolic content present in these foodstuffs is greatly dependent on the cultivar, the harvesting season, and the geographical origin. This fact has allowed the use of the distinct phenolic profiles as 'markers' to discriminate and characterize the diverse geographical areas, with enough potential as to be used for controlling PDO’s (Protected Designations of Origin) [20,51,52,53]. Effectively, according to the location, the phenolic profile did not show any qualitative differences, whereas in terms of quantitative composition statistical differences were observed by several authors in a wide number of phenolic compounds, namely for oleuropein algycone, hydroxytyrosol, tyrosol, lignans, luteolin, apigenin, and for the acids quinic, vanillic, syringic, elenolic, *p*-coumaric, *m*-coumaric, and cinnamic [20,51,52,53,54].

### 2.6. Pathogen Attacks

The olive tree is affected by more than fifty diseases that diminish the yield and quality of olive fruits and, therefore, the stability of olive oils. The verticillium wilt (*Verticillium dahliae*), the olive fly (*Bactrocera oleae*), and the olive anthracnose (*Colletotrichum acutatum* and *Colletotrichum gloeosporioides)* are some of the major drawbacks associated with many olive growing areas [55,56,57].

In Portugal, *C. acutatum* (J.H. Simmonds) is the dominant species, being responsible for more than 97% of incidence, especially in the Alentejo region (southern Portugal), even though both *C. acutatum* and *C. gloeosporioides* cause olive anthracnose in countries of the Mediterranean Basin [58,59]. In wet conditions and in orchards dominated by susceptible cultivars, the pathogen can affect the entire production [60].

Therefore, high economic losses occur as a consequence of the tree death and yield reduction associated with *C. acutatum* infection, which may also entail lower olive oil quality with respect to physico-chemical (oil colour (red), acidity, and stability) and sensory characteristics [60,61]. In addition, this fungus also affects the concentration of phenolic compounds, decreasing their concentration in oils produced from infected drupes [62,63]. However, the incidence of olive anthracnose depends strongly on the cultivar susceptibility, climatic conditions, and pathogen virulence [59]. The tolerance is normally the result of several genes interaction, even though this mechanism still remains poorly understood nowadays. Actually, some cultivars, such as Galega Vulgar (the dominant Portuguese cultivar, accounting for around 30% of Portuguese olive oil production), are more susceptible to the anthracnose than others, like ‘Cobrançosa’ and ‘Picual’, which are moderately-tolerant and tolerant to *C. acutatum*, respectively, while it has been shown that these cultivars, with distinct susceptibilities, display distinct phenolic profiles and quantities [64]. In this sense, it has been observed by Gouvinhas et al. (2016) that Galega Vulgar presents lower quantities of phenolics, respecting the other cultivars mentioned, while these contents increase with the evolution of the time of exposure to the infection, reaching the quantities presented by the two other cultivars assessed at the end of 144 h of exposure [64]. This fact displays the clear correlation between phenolic contents and susceptibility to diseases, being shown that cultivars with higher contents are rather resistant, while the production of this kind of compounds is enhanced by external aggressions. Moreover, the decrease of phenolic contents' synthesis in the final stages of maturation, also led all the cultivars to be more susceptible to this disease in the final ripening stages, further supporting this observation [64].

## 3. Olive Oils

Virgin olive oil is mainly constituted by triacylglycerols, besides other minor compounds, which include almost 230 different chemicals [65]. Glycerols of olive oils, the most characteristic fraction, include a high proportion of mono and polyunsaturated fatty acids, contributing to reach an adequate lipid profile in plasma after dietary intake [66]. In addition, when obtained exclusively through mechanical means, olive oils represent a valuable source of non-nutrients (bioactive phytochemicals), which are partially responsible for the biological activity attributed to this food matrix, being mainly represented by tocopherols and phenolic compounds, largely associated with particular sensory properties, besides biological activity [67]. Since the phenolic content depends on the olives used (varying with agronomical factors, exposure to stress, cultivar, etc.), not all olive oils present similar phenolic composition. Therefore, some of the activities that have been observed in vivo, for EVOO, or their phenolic extracts, were not observed, or were severely decreased [7].

### 3.1. Non Phenolic Compounds of Olive Oil

Since the virgin olive oil is not subjected to refining procedures, this product maintains important compounds that can be classified in saponifiable and unsaponifiable molecules. The saponifiable or glyceride fraction represents from 90.0% to 99.0% of total oil weight and is mainly composed by the phospholipids, mono-/di- and triacylglycerols, whilst the unsaponifiable or non-glyceride fraction (0.5–1.5%) includes hydrocarbons, aliphatic alcohols, sterols, pigments, and several volatile and phenolic compounds.

The saponifiable fraction is mainly composed by triacylglycerols, whilst the diversity of fatty acids present in olive oil includes a number of monounsaturated and polyunsaturated fatty acids including myristic (C14:0), palmitic (16:0), palmitoleic (C16:1), heptadecanoic (C17:0), heptadecenoic (C17:1), stearic (C18:0), oleic (C18:1), linoleic (C18:2), linolenic (C18:3), arachidic (C20:0), eicosenoic (C20:1), behenic (C22:0), and lignoceric (C24:0) acids. From these, oleic acid (C18:1, *n*-9) and linoleic acid (C18:2, *n*-9, *n*-12) are the main components, representing from 55 to 83% and from 5 to 15% of the total fatty acids, respectively. On the other hand, the olive oil composition displays low quantities of saturated fatty acids (8.0–20.0%) [14,68].

The olive oil profile, concerning the content in distinct fatty acids, represents a relevant issue, providing valuable information that allows to distinguish the various classes of oil, such as extra virgin, as well as lampant from virgin oil. Also, within the saponifiable group, minor components such as monoglycerides and diglycerides can be found. These compounds are present in olive oil in small amounts (less than 0.3% and 2.8%, respectively) and are due to the enzymatic hydrolysis of triacylglycerols and incomplete triacylglycerol biosynthesis [69].

Regarding the unsaponifiable fraction, the analysis of these components allowed to describe the following classes:

### 3.2. Hydrocarbons

This class includes the less polar compounds, whilst several studies have reported the presence of elements of the n-alkane series sizing from C_10_ to C_35_ [67]. Within this group, one important compound is noteworthy in virgin olive oil: squalene. Squalene (2,6,10,15,19,23-hexamethyl-2,6,10,14,18,22-tetracosahexaene) is a natural occurring terpenoid in olive, representing the main component of the hydrocarbon fraction (more than 90%). To date, besides its role as valuable dietary component constituting a precursor in cholesterol biosynthesis, squalene has been used as a moisturizing or emollient agent in cosmetic preparations, being also recognized its potential as oxidation inhibitor, exerting a valuable contribution to the stability of olive oil [70,71].

### 3.3. Aliphatic Alcohols

This is a group of natural compounds, which occur in olive oil either free or in the sterified form [72]. The most important are fatty alcohols and diterpene alcohols, while also benzyl esters of hexacosanoic and octacosanoic acids have been also found in olive oil [73]. The latter compounds have focused a growing interest regarding the treatment of a range of chronic diseases because of their biological activity [74]. Besides aliphatic alcohols, some aromatic alcohols are also found, such as benzyl alcohol and 2-phenylethanol, belonging to the olive oil volatile fraction, which also comprises alkanols and alkenols with less than ten carbon atoms in their structure.

### 3.4. Sterols

These are important lipids, with amounts ranging from 855 to 2185 mg kg^−1^, being related to the quality of the oil and broadly used for checking its genuineness. The main sterols present in olive oil are β-sitosterol, campesterol, stigmasterol, clerosterol, sitostanol, and δ-5-avenasterol [75]. These components present a similar chemical structure to cholesterol excluding the addition of an extra methyl or ethyl group, which reduces the cholesterol absorption and thus reducing the circulation levels of cholesterol. Furthermore, the beneficial physiological effects of sterols could be enhanced by the combination with other beneficial substances, such as olive oils [75].

### 3.5. Pigments

Two types of natural pigments, namely, chlorophylls and carotenoids, are responsible for the colour of olive oil. The former compounds, chlorophylls, contribute for the greenness of vegetable oils, whilst carotenoids account for their yellowness. The structure of chlorophyll pigments consists of one tetrapyrrole macrocycle coordinated to a Mg^2+^, containing an additional isocyclic ring. This structure provides a very stable planar complex, representing a chromophore of several conjugated double bonds, responsible for the absorbance in the visible region of the spectrum, featured by these pigments [76]. The hydrophobic nature of these pigments is due to the presence of an esterified phytol group. In some olive oils, these compounds exist as both chlorophyll-a and chlorophyll-b, being described the presence of a methyl group in C3 in the former, which is substituted by an aldehyde group in chlorophyll b. The ratio between chlorophyll a and b in this foodstuff ranges from 6 to 8 [77].

Additional pigments described in vegetable oils are represented by carotenoids, which account for many biological properties and specific activities of this kind of unsaponified compounds [78]. Most of the carotenoids described display 40 carbon atoms, and can be classified as cyclic or acyclic compounds, depending on the presence or absence of rings in their structures. From the chemical point of view, carotenoids can be divided into carotenes (solely constituted by carbon and hydrogen) and xanthophylls (also containing oxygenated functions, like epoxide, hydroxyl, acetate, carbonyl, and carboxylic groups, among others). In olives, carotenoids can be found free or associated with other primary metabolites (fatty acids, sugars, and proteins) [78,79]. The main carotenoids present in olive oil are lutein and β-carotene.

### 3.6. (Poly)phenolic Composition of Olive Oil

Most of unsaturated fatty acids have been traditionally pointed as the main responsible for the beneficial health effects of dietary olive oil, particularly assigned to oleic acid. However, the absence of beneficial biological activity of some foods displaying a high content in these saponifiable compounds, raised the question of the potential contribution of additional compounds to the health benefits provided by olive oil. Currently, major attention has been paid on polyphenolic compounds present in olive oil. These compounds display a great structural diversity, as well as a plethora of biological functions in plants, including the contribution to pollination, plant defence systems against biotic and abiotic stress, and tissues structure maintenance and pigmentation [80].

In olive oil, simple and complex phenolic compounds contribute to its stability regarding the oxidative status, besides substantially affecting its sensory properties. Therefore, an interest in olive oil as a valuable source of these bioactive compounds has emerged, boosted by the experimental data on their benefits for human health, which has been supported by a number of in vitro and in vivo assays. The most abundant phenolics present in olive oils can be classified according to diverse criteria, while the most widely accepted organization of phenolics in olive oil allows to distinguish the following types: cinnamic and benzoic acids, phenolic alcohols, secoiridoids, lignans, hydroxy-isochromans, and flavonoids [81]. These categories, which constitute the polar phenolic fraction of olive fruits and olive oil, comprise around 45 individual compounds (Table 1).

The differences between phenolic compounds are based on the number of rings and their aromaticity, so as to the elements bonded to these structures, which sometimes are connecting distinct phenolic moieties. The polar phenolic compounds of olive oil are obtained by liquid-liquid partition using hydromethanolic solvents. These compounds contain one or more hydroxyl groups (-OH) directly attached to an aromatic ring, and their structures range from that of a simple phenolic molecule, such as phenolic acids, to complex high-molecular mass oligomers, such as tannins [82]. The class “polar phenolic compounds” is used to differentiate them from lipophilic phenols, where tocopherols and tocotrienols are included.

Some components of this latter group of phenols are also found in other vegetable oils, representing α-tocopherol almost 90.0% of the total content in tocopherols [83].

The majority of the naturally occurring phenolics in plant foods are found as conjugates with mono- and polysaccharides, which appear conjugated to one or more of the hydroxyl radicals, and may also occur as functional derivatives such as esters and methyl ester [84]. The classification of phenolic compounds in polar and non-polar has a further interest to understand the extraction ratio from the food matrix during the digestion process and the further bioavailability (absorption, organic distribution, and excretion).

### 3.7. Benzoic and Cinnamic Acids

Phenolic acids are the first group of phenols discovered in olive fruits and virgin olive oils [98], being divided in two distinct groups, which display the chemical structures C_6_–C_1_ and C_6_–C_3_, corresponding to benzoic and cinnamic acids, respectively (Table 1). The former group includes gallic, vanillic and syringic acids, while caffeic, ferulic and sinapic acids, which constitute some of the most important phenolic cinnamic acid derivatives [99]. Another abundant hydroxycinnamic acid derivative is verbascoside, which is present in peel, pulp and seed. This is a disaccharide comprising glucose and rhamnose bounded to a hydroxytyrosol and hydrocycinnamic acid molecule, respectively [95]. This group of these compounds are synthesized by the general phenylpropanoid pathway that, as the name indicates, generates a common substrate to a number of phenylpropanoid compounds, including the hydroxycinammic acids. This process starts with phenylalanine, generated via the shikimate pathway, which is the deaminated, resulting in cinnamic acid [100,101].

### 3.8. Phenolic Alcohols and Secoiridoids

Hydroxytyrosol (3,4-(dihydroxyphenyl)-ethanol) and tyrosol ((*p*-hydroxyphenyl)-ethanol) are the most abundant phenolics belonging to this group. Nonetheless, these compounds are found at low concentration in fresh oils, increasing their proportion during storage, since they result from the hydrolysis of secoiridoids present in olive oil, which contain them in their molecular structures [102]. Oleuropein is the most abundant secoiridoid, being one of the three major phenolic compounds found in olive oil, alongside the phenolic alcohols hydroxytyrosol and tyrosol, these three compounds representing up to 90% of the total phenolic content [103]. Secoiridoids, including oleuropein, are generated during the oil mechanical extraction process by endogenous β-glucosidases, which catalyse the hydrolysis of oleuropein, demethyloleuropein, and ligstroside [104]. In olive oil, oleuropein is present in the aglycone form (Table 2).

Hydroxytyrosol, tyrosol, and oleuropein are related structurally. The only difference between hydroxytyrosol and tyrosol is the presence of a hydroxyl group in the *meta* position in the former molecule, while oleuropein consists on the di-hydroxytyrosol ester derivative of elenolic acid. During ripening, the concentration of oleuropein in olive fruits decreases, whilst its hydrolysis product, hydroxytyrosol, increases in a proportional manner [105].

### 3.9. Lignans

Phenolics within the lignan group were firstly identified in olive oil by Brennes and others [102], which isolated and identified (+)-pinoresinol and (+)-1-acetoxypinoresinol (Table 2). These compounds are present in olive pulps and the woody portion of the seed, undergoing marginal biochemical modifications during the mechanical extraction, whilst major changes of the concentrations of these compounds are due to distinct agro-environmental conditions [102].

### 3.10. Hydroxy-Isochromans

Hydroxy-isochromans compounds were identified in olive oil by Bianco et al. [106]. These compounds are formed during the olive oil extraction due to the malaxation process, which leads to the occurrence of the compounds involved in the formation of isochroman derivatives. Thus, hydroxy-isochromans are synthesized by condensation of hydroxytyrosol and aromatic aldehydes, which finally results in a planar bi-cyclic structure comprising one aromatic and one saturated ring, the latter encompassing an oxygen atom in the structure, while the hydroxyl groups are those that were previously present in the hydroxytyrosol molecule (Table 2) [106].

### 3.11. Flavonoids

Some flavonoids are also present in olive oils (Table 2) [107]. Flavonoids are low molecular weight compounds with a C_6_–C_3_–C_6_ structure, whilst this designation encompasses diverse compounds. Within the flavonoids molecular structure, the aromatic ring A is derived from the acetate/malonate pathway, whilst the B ring results from phenylalanine through the shikimate pathway [108]. Luteolin may be originated from rutin or luteolin-7-glucoside, and apigenin from apigenin glucosides. Besides these two compounds, also quercetin, in its simple and glucosilated forms has been found in olive oils [109].

### 3.12. Lipophilic or Non-Polar Phenols

These phenolic compounds are mainly represented in olive oils by tocopherols and tocotrienols, which are heteroacids of high molecular weight. α-tocopherol is the most abundant (90.0%), although β and γ-tocopherols are also present [110]. The percentages of tocopherols in olive oils are critically influenced by a number of factors, representing a very relevant criterion of purity [111].

Besides the phenolics described, other phenols with diverse molecular structures have been identified, such as oleoside-11-methylester [88] and 3,4-dihydroxyphenylglycol [97].

## 4. Chromatographic and Spectroscopic Methods for the Determination of Olive Oil Composition

The wide diversity of compounds described in olive oil has been identified and quantified resorting to currently applied instrumentation and analytical techniques. These include spectrophotometric methods, gas chromatography (GC), and liquid chromatography (LC) coupled with ultraviolet (UV), and photodiode array (PDA) and mass spectrometer (MS) detector. Furthermore, Nuclear Magnetic Resonance (NMR) spectroscopy, as well as mass spectrometry have been also applied to obtain additional information for the accurate identification of individual compounds in complex food matrices. The evolution of analytical techniques over the last years have allowed to update data regarding the qualitative and quantitative composition of olive oils, which exposed inconsistencies regarding the concentrations of phenolic compounds found, mainly due to the employment of diverse analytical methodologies presenting divergent responses. Furthermore, besides the well-established NMR, other spectroscopic techniques, namely vibrational, such as Infrared (IR) and Raman spectroscopy have been explored for the assessment of these contents [116].

Spectrophotometric methods have been extensively used for the determination of the total phenolic content in plant foods (including vegetable oils) as well as in separate classes of phenolic compounds, since they do not require expensive equipment. However, these analytical procedures enclose diverse constraints related with the limited information provided, the overestimation of the phenolic concentration and the lack of qualitative information on individual bioactive phenolics [117]. In olive oil, there are several phenolic extraction methods employed before the total phenolic content analysis, mainly based on liquid/liquid extraction (LLE) and solid phase extraction (SPE) methodologies, using essentially methanol as solvent. In the first case, the phenolic fraction of olive oil can be isolated with methanol [103] or with methanol/water, being the use of methanol/water 80:20 (*v/v*) one of the most reported and efficient solvent [98]. However, some authors demonstrated a complete recovery of this components using 100% of methanol as an extraction solvent. Concerning the SPE technique, some authors investigated the recovery of phenolic compounds by the use of commercial available solid supports, such as octadecyl (C_18_) and octadecyl end capped (C_18 EC_), revealing higher recovery when the C_18_ sorbent phase was employed, demonstrating also that this methodology can be highly competitive with the liquid/liquid extraction procedure [86].

### 4.1. High Performance Liquid Chromatography

This technique represents a sensitive and specific method that allows overcoming the limitations previously pointed to colorimetric methods, especially when coupled to MS instrumentation, which provides valuable information for the identification of individual compounds [118]. Besides these classical analytical approaches, over the last years, other methods have been developed, namely, enzymatic methods [119] and a combination of MS with atmospheric pressure chemical ionization [120]. Nonetheless, high performance liquid chromatography (HPLC) constitutes the most widely used technique for the separation and quantification of phenolic compounds in different food matrices, including olive oil (Table 3).

When considering HPLC instrumentation, diverse alternatives can be emphasized. In this sense, the reversed phase HPLC has become a dominating tool to separate and identify individual phenolics by diverse detection systems, such as diode array detector (DAD), or tandem MS. Recently, an increasing number of LC methods are being developed, not only regarding the assessment of the phytochemical composition, but also to improve the separation of phenolic compounds’ efficiency and to reduce costs, besides improving sensitivity [121].

Some of these features have been reached with new experimental setups, such as ultra-high performance liquid chromatography-electrospray ionization source-MS/MS (UHPLC-ESI-MS/MS) [122], rapid resolution LC-MS [123], LC-Electrospray ionization (ESI), Time of flight (TOF)-MS [54,124], and HPLC-DAD-FLD system [123]. These approaches use narrow-bore columns packed with very small particles (1.8 μm) and high flow rate with delivery systems operating at high back-pressures. Concerning the detectors, the most used are the diode array (DAD) and the mass spectrometer (MS), and recently, the fluorescence detector (FLD) has been also employed [123].

### 4.2. Spectroscopic Methods: IR and Raman

Besides NMR, a spectroscopical technique widely used for structural determination of compounds, such as the ones isolated through preparative-HPLC, other spectroscopical techniques have been lately explored for the assessment of olive oil and olives. Among these, vibrational techniques, such as IR, generally Fourier Transform-IR (FTIR), and Raman, have emerged recently as analytical methods widely used for food and feed analysis [125].

Actually, the conventional measurements employed in the assessment of the chemical composition of olive oils, such as peroxide values, free acidity, fatty acids, phenolic and volatile compounds, are time consuming and require large amounts of reagents and solvents, which are toxic and expensive. Furthermore, they require the pre-treatment of sample. On the other hand, spectroscopical means, such as FTIR—particularly when used in conjunction with Attenuated Total Reflectance (ATR)—and Raman spectroscopy, dismiss any kind of sample preparation, avoiding the occurrence of chemical transformations, such as oxidation [126,127]. Furthermore, while the ATR module works within the medium-IR range (MIR), there are other accessories available, such as Diffuse Reflectance Integrating Sphere-FT (DRIFT), which, besides also dismissing the sample preparation, register the samples’ spectrum within the near-IR (NIR) range, thus retrieving supplementary information [116].

These vibrational spectroscopic methods provide information about the chemical composition of various food and biological materials, and molecular structure. Both Raman and IR assess the same physical property—molecular vibrations—but present different selection rules, retrieving complementary information [134]. In the last few years, the application of IR and Raman spectroscopy, coupled to chemometric data analysis methods, has been implemented for authentication, determination and evaluation of the composition of olive oils [135]. Furthermore, this combined methodology has been successfully used to assess promptly the contents in total phenols, *ortho*-diphenols and flavonoids in both olive oil and olives, thus, retrieving the hot prospect of evaluating specific phytochemicals in these matrices, resorting to this methodology [126,136].

## 5. Relationship between Molecular Structure and Biological Activity of Olive Oil Phenolics

The oxidation reactions play an important role, not only in terms of human physiology, but also in food industry. In the human organism, the oxidation reactions may lead to oxidative stress, which is responsible for some oxidative damage of DNA, proteins, and other molecules, and may lead to some human diseases, such as atherosclerosis, cancer and degenerative diseases (Parkinson and Alzheimer diseases) [137]. In food industry, these reactions are associated to rancidity, enzymatic browning, and oxidative spoilage of fruits, vegetables and beverages.

Within the body, there are several antioxidant systems resulting from regular metabolic processes that can attenuate oxidative stress, while the cell-damaging effect of free radicals can be also inhibited by the antioxidants present in diet, since these can be complementary for the endogenous system. In virgin olive oil, the resistance to oxidation is mainly attributed to the content of fatty acid composition and minor compounds, including tocopherols, polyphenols and chlorophylls. However, in this vegetable oil, the main antioxidants are the polyphenols [12]. In the last few years, many reports demonstrated biological activities of olive oils, such as antioxidant, anti-inflammatory, analgesic, and antimicrobial activity, supporting the use of phenolic compound in pharmaceutical, food, and cosmetic industries [138,139]. The antioxidant activity of phenolics have been related to a lower incidence of coronary heart disease, lower risks of some type of cancers—since they can reduce DNA damage, reduction of lipid peroxidation and the amount of reactive oxygen species (ROS) generated, diminished inflammation and the inhibition of platelet-activating factor [16,101].

Phenolic compounds are important for the olive oil stability and protection against the oxidation that can occur during storage. Thus, there is a relationship between the phenolic content and the oxidative stability of olive oil [140]. Natural antioxidants present in olive oil are the main responsible for its shelf-life once they inhibit oxidation processes. Despite being rather complex to found concrete relationship between the molecular structure of olive oil phenolic compounds and their possible health effects, some authors reported valuable information concerning this issue. Structure–activity relationship (SAR) studies are normally carried out by making minor changes to the structure of a lead compound to produce analogues, thus assessing the impact of these structural changes on biological activity. The most important antioxidant activity of olive oil phenolic compounds is related to the free radical-scavenging ability, by stopping the propagation chain during the oxidation process through the donation of a radical hydrogen to alkylperoxyl radicals (produced by lipid oxidation) and the formation of stable derivatives during this reaction. Phenols can also act as metal (Fe^2+^, Cu^2+^) chelators, preventing their involvement in Fenton reactions that can generate high concentrations of hydroxyl radicals [141]. For example, caffeic acid is able to block the increase of the concentration of Ca^2+^ in response to lipoprotein oxidation [142]. These activities allow these compounds to interact with biological systems, preventing degenerative diseases linked to oxidative stress in separate tissues and organic systems.

Simple phenols, secoiridoids and lignans present antioxidant activities and properties. For example, compounds possessing an *ortho*-diphenolic structure display high antioxidant activity, due to the improved radical stability through the formation of intramolecular hydrogen bonds formed during the reaction with free radicals. Furthermore, the O-H bond of phenol is weakened by electron-donating substituent in the "ortho" position, thus, facilitating the formation of the phenoxyl radical [143]. Therefore, hydroxytyrosol, oleuropein aglycon and decarboxymethyloleuropein aglycon present better radical-scavenging capacity than single hydroxyl substitutions, as in the case of tyrosol, which provides no activity and, thus, does not protect low density lipoproteins (LDL) from oxidation [144]. The high antioxidant activity of hydroxytyrosol is also due to its reducing power on Fe^3+^ [145]. Oleuropein presented slightly weaker radical scavenging activity than hydroxytyrosol by the DPPH and ABTS methods [118]. Gallic acid, which possess three hydroxyl groups, is also one of the most potent scavengers, due to the pyrogallol structure (3,4,5-OH), corresponding to a noticeable H-donating ability. This high antioxidant activity due to the presence of hydroxyl groups in *ortho* position, was proven with radical scavenging tests and Rancimat test [145,146,147]. Furthermore, Finotti and Di Majo demonstrated that the *ortho*- and *para*- substitutes of the radicals are more stable than the *meta*- ones [148]. Moreover, the antioxidant activity of phenolic acids increases with increasing degree of hydroxilation, while the substitution of the hydroxyl groups at the 3- and 5-position with methoxy groups, such as in syringic acid, reduces the activity [149].

The –COOCH_3_ fragment, present, for instance, in oleuropein aglycon, seems to cause a decrease in the antioxidant activity, which is related to the inability of this group to function as H-donor [87]. On the other side, Visioli and others [150] reported that oleuropein have the capacity to inhibit LDL oxidation of free radical scavenging [150]. Hydroxycinnamic acids, such as ferulic, sinapic, caffeic, chlorogenic and *p*-coumaric, displayed higher antioxidant capacity than the hydroxybenzoic acids, like *p*-hydroxybenzoic, syringic and vanillic acids [151,152]. The –CH=CH–COOH group linked to the phenyl ring of hydroxycinnamic acids, confers higher radical stabilization, due to the enhanced electronic delocalization, resulting in higher H-donating ability respecting the –COOH group present in hydroxybenzoic acids [76,150,152].

For flavonoids, displaying more complex structures, the SAR’s are rather intricated. In fact, some structural groups are important for determining their radical scavenging ability: the -OH groups, namely the *ortho*-di-hydroxyl group, in the B-ring, the 2,3-double bond conjugated with the 4-oxo function in the C ring, the 2,3-double bond combined with a 3-OH in the C ring, the presence of both 3- and 5-hydroxyl groups together with the 4-oxo function in the benzopyran moiety for maximal radical-scavenging capacity and enhanced radical adsorption, and the substitutions of hydroxyl groups in ring B by methoxy groups [153]. Therefore, the diversity of structures possibly found in these polyphenols contributing to free radical-scavenging activity, has shown that the ensemble of all these features can lead these compounds to be more effective antioxidants in vitro than vitamins E and C [150,154]. So, the antioxidant activity of flavonoids does not depend only on the number of hydroxyl groups, but also on the position of these groups and glycosylation and configuration of other substituents [155].

Concerning the radical scavenging capacity of lignans, to the best of our knowledge, no studies related to this determination in olive oils are available, while some authors reported that although not possessing an *ortho*-dihydroxy structure, they present some activity, but very low [154,155,156].

Generally speaking, it can be pointed a lack of reliable knowledge regarding a thorough understanding of the SAR’s displayed by each family of compounds, concerning the biological activities that they have been related to. Therefore, it would be desirable the development of comprehensive studies for the most important families of compounds, such as secoiridoids, with the view of evaluating the impact of distinct substitution patterns in the biological activities displayed, thus allowing the attainment of the structural features behind these activities.

## 6. Biological Activity of Olive Oil Compounds: In Vitro and In Vivo Evidence

**Figure 2:**
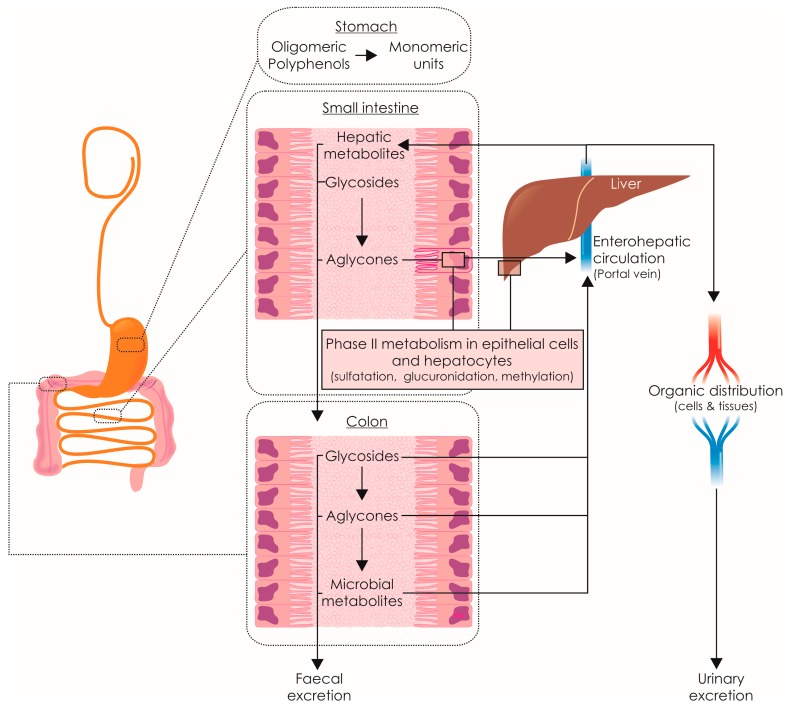
Scheme of the routes involved in the bioavailability and excretion of dietary phenolic compounds.

The bioavailability of a phenolic compound is referred to the degree in which it is liberated from food and absorbed in the intestinal tract. The highest plasma concentration of derivatives from olive oil is recorded from 1 to 3 h after ingestion, indicating that the major absorption of these compounds takes place in the small intestine. Once absorbed, phenolics undergo a phase II metabolism in the epithelial cells of the intestine wall and liver. Metabolized and intact molecules are transported by the enterohepatic circulation to the liver, where the initialized transformation is completed. From liver, phenolic derivatives are spread over the organism by blood stream and secreted to the intestinal lumen by bile. Part of the ingested compounds reach the colon where the local microflora produces metabolic derivatives also responsible for the biological activity of phenolic compounds of olive oil (Figure 2).

Although phenolic compounds of olive oil have demonstrated a healthy activity by several in vitro and animal models, when assessing the biological potential of these compounds by human clinical trials and dietary interventions, many of the modulator activities described in vitro were not proven. Thus, it is important to remark that doses assayed in vitro and in experimental animals are frequently higher than those applied in humans and, in addition, the metabolism of phenolic compounds may differ in these diverse models. Thus, the assessment of the actual biological activity of virgin olive oil in vivo requires complementary determinations and long-term interventions in humans to understand the real benefits exerted when regularly included in balanced diets [157].

Actually, one of the most important constraints to fully understand the actual benefits of EVOO consumption, lies on the lack of definitive studies relating serum levels of olive oil phenolics to olive oil intake in human volunteers, which would allow to clearly understand the bioavailability of most of the compounds that have been assessed in vitro. Moreover, polyphenols are dose-dependently absorbed and extensively metabolized mainly as glucuronides. In this sense, their bioavailability varies among the different classes, while plasma concentrations of polyphenols have been reported to be lower than 10 µM, in the range of 0.1–1.0 µM in most cases, although higher concentrations have been reported after virgin olive oil intake in human studies, showing that this foodstuff may represent an actual contribution for these concentrations [158]. However, the average daily intake of phenols from olive oil and their plasma concentration in olive oil-consuming populations is rather unknown, whereas calculations based on a typical Mediterranean diet point to an intake of 10–20 mg/day of total phenols, supplied by olive oil alone. Therefore, it is difficult to estimate the likely physiological concentrations of polyphenols occurring in plasma, and tissues, due to olive oil-consumption [159].

Nevertheless, the presently available data allow to estimate that olive oil presents 14.42 mg hydroxytyrosol kg^−1^ and 27.45 mg tyrosol kg^−1^, corresponding to concentrations of 81.7 µM hydroxytyrosol and 175.5 µM tyrosol, while these data must be carefully considered due to the number of factors that may affect the concentrations of these compounds in olives, and thus, olive oil. Concerning the available publications refering to actual polyphenolic concentrations in plasma, after olive oils intake, it has been reported that the absorption of hydroxytyrosol and tyrosol in human subjects (determined in 24 h urine samples) was 30–60% and 20–22%, respectively, of total intake. Therefore, up to 49 µM hydroxytyrosol and 38.6 µM tyrosol from virgin olive oil might be absorbed, while other authors claim that regarding these compounds, the physiological concentration after oral ingestion of olive oil is in the range of 10–100 µM [160]. Concerning oleocanthal, another compound with special relevance, the daily consumption of 50 g of EVOO containing up to 200 µg per mL oleocanthal would correspond to an intake of up to 9 mg per day, with absorption values of 60–90% being reported for this compound, higher than those for tyrosol and hydroxytyrosol [161]. Furthermore, concerning oleuropein, another molecule with biological relevance, this compound is rapidly absorbed from the intestine, reaching a maximum (peak) of plasma concentration of 370 µM after 2 hours of oral administration of 20 mg kg^−1^ in rats [162].

These studies show that some of the most important bioactive compounds present in olive oil can reach noticeable concentrations in plasma, after intake, pointing to the feasibility of the direct benefits of olive oil consumption, while these concentrations are within the range of the effective concentrations observed in some of the in vitro/in vivo studies developed till the present moment, concerning important biological activities, such as anticarcinogenic (Table 4 and Table 5).

In this sense, there are activities evaluated in vitro, which were observed in significant extents for concentrations that are realistically within the ranges of a dietary intake. For instance, oleuropein, displays biological activity at 15 µM, reducing monocytoid cell adhesion to stimulated endothelium, as well as vascular cell adhesion molecule-1 (VCAM-1) [171]. Furthermore, noticeable nitric oxide scavenge activity has been registered for the same compound in concentrations as low as 1 µM [172], while it has presented relevant anti-proliferative activity against bladder carcinoma cells with an IC_50_ of around 8 µM [158]. Also, hydroxytyrosol, found in relevant concentrations in EVOO, displays the capacity to scavenge hypochlorous acid in neutrophils, with a remarkable EC_50_ of 3.2 µM [173], besides presenting anti-proliferative activity against bladder carcinoma with an IC_50_ of around 10–12 µM [158]. Finally, concerning these compounds, oleocanthal, which is also absorbed in significant extents (60–90%) displayed capacity to inhibit carcinogenesis in concentrations of around 3 µM [168], also presenting the ability to induce apoptotic cell death in human breast and colon carcinomas, besides the inhibition of the metastatic process in human breast cancer cells (MDA-MB-231), at the concentration of 15 µM [168,174]. Besides the bioactive compounds present in olive oil, some of the resulting metabolites can be also observed in noticeable concentrations after oral intake, while some of these bioactive compounds, such as oleuropein aglycone, can also present noticeable activity (Table 4) [105,165].

### 6.1. Oxidative Stress

The production of reactive oxygen and nitrogen species in cells cannot be overcome since they result from several cell functions, namely, energy supply, chemical signalling, detoxification, and immune function. However, an overproduction of these reactive species, an exposure to external oxidant substances, or a failure in the defence mechanisms, entail critical consequences for cells’ molecules (DNA, RNA, proteins, and lipids), causing an increased risk and an early onset of diverse degenerative and disabling pathologies [137]. Therefore, their balance within cells’ compartments needs to be maintained by endogenous enzymes, which are represented by superoxide dismutase, glutathione peroxidase, and catalase. The antioxidant activity of phenolics described in olive oil suggested that diverse compounds may contribute significantly to the antioxidant activity. For instance, oleuropein aglycone di-aldehyde has been stressed as the main responsible for this antioxidant power [105], which is mainly supported by its capacity to scavenge DPPH, ABTS, hydroxyl radical, hydrogen peroxide, and superoxide anion [175]. This was further confirmed by Paiva-Martins and collaborators through the assessment of hydroxytyrosol, oleuropein, and oleuropein aglycone di-aldehyde to protect red blood cells from oxidative damage in vitro [176].

**Figure 3:**
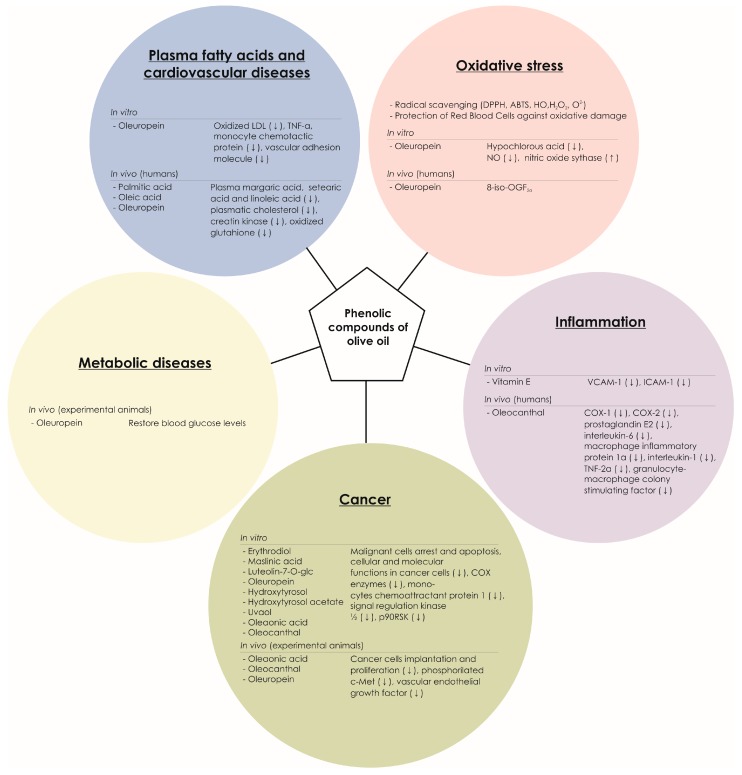
Biological activities of phenolic compounds present in olive oil demonstrated in humans.

Since radical scavenging activity constitutes one of the most relevant activities of phenolic compounds, the capacity of dietary olive oil to modulate oxidative stress markers in vitro and in vivo has been extensively studied in humans. Thus, the antioxidant activity of phenolic compounds has been demonstrated by several in vitro studies that have shown the potential of oleuropein to scavenge hypochlorous acid in neutrophils isolated from health volunteers [173]. This compound is related to inflammation, which indicates the capacity of olive oil phenolics to influence oxidative pathways associated with diverse health claims. Also in vitro, oleuropein has been also proven on its ability to scavenge nitric oxide and to promote the expression of the inducible nitric oxide synthase in cells [172]. Although it has not been further proved in humans, the assessment of the radical scavenging capacity of oleuropein, in vitro, has evidenced its ability to form intramolecular hydrogen bonds, in the catechol moiety, between the free hydrogen of the hydroxyl group and its phenoxyl radical [177], which could support the antioxidant activity demonstrated in vivo (Figure 3). Furthermore, Visioli and others [177] demonstrated in healthy volunteers that dietary oleuropein decreases the urinary excretion of 8-iso-PGF2α, which indicates lower in vivo peroxidation of lipids (Table 4 and Table 5) [163].

Besides phenolic compounds, pigments present in olive oil (chlorophyll and carotenoid derivatives) have been emphasized on their beneficial effect on human health after dietary intake. Some controversial data showing prooxidant and antioxidant [184] activity of chlorophyll derivatives have been reported, while it is well established that even compounds exerting noticeable antioxidant activity may act as prooxidants, depending on their concentrations [185]. Furthermore, no evidences of any of both activities have been demonstrated in vivo neither by animal models nor in human trials. In the same way, other pigments, carotenoids, have been pointed as effective antioxidants [186,187], even though some controversy still remains, regarding their activity in vivo, because of the lack of dedicated evaluations and the description of prooxidant activity under certain conditions [188,189]. Nevertheless, there are effects that can be either assigned to the consumption of whole olive oil, or to quantities of specific compounds comparable to the concentrations in which these might be found in EVOO. For instance, a significant protection against induced oxidative stress was observed in rat hearts pre-treated with as little as 20 mg kg^−1^ of oleuropein, a dose comparable to the average daily intake of biophenols from olive oil in the Mediterranean diet (Table 5) [166].

Another issue, concerning in vivo studies, for the evaluation of specific compounds which are actually contributing to the health benefits observed, lies on the difficulty to evaluate the contribution of each specific compound, since the olive oils are sometimes supplemented with specific compounds [165], thus, difficulting the comparison between the results of these studies and the actual consumption of whole olive oil, while it is of the utmost importance the knowledge of the plasmatic levels of the compounds or metabolites, in the same studies, so these can be rationally associated to the benefits observed.

### 6.2. Cancer

Concerning the potential of dietary olive oil to reduce the incidence, severity, and progression, of cancer processes in humans, a number of epidemiological studies have indicated a lower incidence of certain types of tumors in the Mediterranean basin, which has been attributed to the influence of the ingredients included in the Mediterranean diet [190] as demonstrated by the Lyon Diet Heart Study, which showed a reduction of cancer risk and mortality of 61 and 56%, respectively [191]. An important contribution to these numbers can be assigned to dietary olive oil, which constitutes an essential ingredient in the Mediterranean diet, providing a high amount of monounsaturated fatty acids and bioactive phytochemicals capable of inducing beneficial effects on health [192].

The anti-cancer activity of dietary olive oil in humans (in vivo) has been mainly attributed to the capacity of its nutritional and non-nutritional components to inhibit the onset of the cancer process and its progression at different stages by protecting against oxidative DNA damage, modulation of biosynthesis of the colon cancer promoters bile acids, decreasing estrogen synthesis in adipose tissue, antistrogen effect by structural competition, decrease of free estradiol, changes in cell membrane fluidity, structure, and degree of peroxidation, modulation of genes involved in cell proliferation, and anti-inflammatory and immunomodulatory effects, which were extensively reviewed by Escrich et al. (2007) [193].

The anti-tumor properties of bioactive compounds present in olive oil have been demonstrated in vitro against diverse human malignant cell lines, namely, HT-29 (colon adenocarcinoma), MCF-7 (breast adenocarcinoma), urinary bladder carcinoma, and hepatocellular carcinoma (HepG2) cell lines, being so far proven the capacity of erythrodiol and maslinic acid (triterpenoids) to induce arrest and apoptosis in colon adenocarcinoma cells (HT-29) [17,179]. The activity of terpenoids has been assigned to their capacity to disrupt structural elements, preventing the maintenance of a viable cell structure and functions, thus inhibiting the cell growth by G1 cell cycle arrest [194]. Also in vitro, an additional work has shown the capacity of the bioactive compounds erythrodiol, uvaol, oleanolic acid, and maslinic acid to inhibit cellular and metabolic functions of breast carcinoma cells [178]. Luteolin-7-*O*-glucoside, oleuropein, hydroxytyrosol, and hydroxytyrosol acetate also have been assessed regarding their capacity to inhibit malignant cells proliferation through in vitro assays performed on breast and urinary bladder carcinoma cells (lines MCF-7 and T-24), which allowed to observe only a residual anti-tumoural effect in vitro for these compounds [179] (Table 4 and Table 5).

In fact, even though the capacity of phenolic antioxidants of olive oil, such as hydroxytyrosol and tyrosol, to inhibit oxidative stress after its dietary consumption further contributes to protect against cancer [113], the efficiency of these compounds differs depending on the target cell type. In this connection, hydroxytyrosol and tyrosol induce an increase of the radical oxygen species level in breast epithelial cells. These results point out that hydroxytyrosol and tyrosol attenuate oxidative stress in normal cells, preventing the malignization by protecting against DNA damage [183], although they are inefficient when the malignancy has occurred.

Besides the cytotoxic effect demonstrated in vitro against cancer cells, oleuropein and hydroxytyrosol display antiangiogenic activity [195] inhibiting the creation of the surrounding interacting network that provides the microenvironment required for the malignant cells growth, migration, and invasion [192]. Oleuropein has been also stated as the main responsible for the anti-tumour activity of olive oil by in vivo assessments with experimental animals, regarding the prevention of skin and breast cancer [113,164]. Thus, the preventive effect of oleuropein on chronic UVB-induced skin regarding carcinogenesis and tumour progression may be due to the inhibition of the expression of vascular endothelial growth factor and metalloproteinase 2, 9, and 13 through the reduction of COX-2 level [158], while regarding breast cancer, oleuropein was efficient in minimizing the tumor size, being able to completely remove 9–12 days tumors by disturbing the actin cytoskeleton of tumor cells in vivo [193].

Nevertheless, to the present date, results on the cytotoxic activity of olive oil phenolic compounds recorded in vitro, have been only partially reproduced in vivo because of the metabolic transformation of these compounds after absorption, which entails the exposition of malignant cells to lower concentrations in vivo respecting in vitro models. In this connection, the anti-tumour activity of oleanolic acid against hepatocarcinoma cells has been recently evaluated in vitro and in vivo (mouse model) [167]. In this procedure, after the transfer of HepG2 cells subcutaneously to mice, oleanolic acid was efficient in the inhibition of the cancer cells implantated, preventing the cells proliferation and inducing apoptotic cell death both in vitro and in vivo (Table 5) [167].

Other bioactive compound present in olive oil, oleocanthal, has been identified as a potent COX (cyclooxygenase) inhibitor, which turns it into a valuable candidate to be tested regarding anti-cancer activity. Among the biological functions demonstrated in vitro for oleocanthal against cancer cells, it has been stressed the attenuation of monocyte chemoattractant protein 1, which is a critical instigator of malignant lesions [182]. Moreover, oleocanthal displays an anti-proliferative effect via the inhibition of extracellular signal-regulated kinases 1/2 and p90RSK phosphorylation, promotes cell apoptosis by activating caspase-3 and PARP, and induces DNA fragmentation in human malignant cells [168]. Additionally, in vivo, oleocanthal displayed an inhibitory effect on migratory and invasive actions characteristic of cancer cells, responsible for the metastatic process, possibly as a result of its ability to inhibit c-Met phosphorylation [174] (Table 5).

Despite the cumulating evidences on the anti-tumour activity of bioactive compounds of olive oil in vitro, the physiological concentrations of these compounds constitute a major constraint to extrapolate the cytotoxic effect observed in vitro to in vivo models. For instance, regarding hydroxytyrosol and tyrosol, the assessment of the cytotoxic effect at physiological concentration after oral ingestion concentrations in vitro did not show any effect on cells proliferation, requiring concentrations from 2 to 10 folds higher than the peak concentrations reached after oral administration [160,196]. Nevertheless, these results have to be accounted cautiously, since, even though hydroxytyrosol and tyrosol attenuate oxidative stress in normal cells, protecting the DNA, their efficiency when the malignancy has occurred is limited [181].

### 6.3. Plasma Fatty Acids Composition and Cardiovascular Diseases

Plasma fatty acids concentration and hypertension have been identified as the major risk factors regarding heart diseases. Hence, several epidemiological studies have evaluated the capacity of dietary olive oil to modify the plasma fatty acids profile. Mayneris-Perxachs and others [197], within the framework of a randomized controlled trial design, compared the effect of the Mediterranean diet supplemented with virgin olive oil (PREDIMED Study) on plasma fatty acids, regarding metabolic syndrome, after 1 year of intervention. The results obtained evidenced that the dietary supplementation with olive oil increases the plasma concentrations of palmitic and oleic acids, whilst lowering the level of margaric, stearic, and linoleic acids [197]. These results reinforced previous studies on the effect of diets enriched with monounsaturated fatty acids, which also reported an augment of plasma palmitic acid and decrease of stearic acid level [198], while these modifications, regarding plasma fatty acids, have been related to the risk of several cardiometabolic diseases [198,199]. According to the correlation analysis performed by Maneris-Perxachs and others [186], plasma concentration of oleic acid is a good biomarker of olive oil consumption, since it represents 70–80% of its monounsaturated fatty acids.

An additional factor, to be considered for the prevention of cardiovascular diseases, is the degree of oxidation of plasma lipoproteins. Thus, regarding the capacity of olive oil to protect against these events, oleuropein appeared as capable to diminish low-density lipoprotein (LDL) oxidation both in vitro, inhibiting LDL copper-induced oxidation [159], and in vivo, reducing plasmatic levels of total, free, and ester cholesterol in rabbits [165]. In addition to the prevention of lipoproteins oxidations, oleuropein was efficient regarding the protection against heart damage induced by ischemia reperfusion in rats, the pre-treatment with oleuropein leading to a reduction of creatine kinase and oxidized glutathione release, which are markers of myocardial damage, during the perfusion of this organ [166]. Oleuropein has been also stressed on their role as anti-thrombotic and anti-atherogenic agent, although this property depends on its anti-inflammatory and anti-oxidative activities [166]. Moreover, another activity assigned to this compound, the inhibition of the atherosclerosic process, is due to the capacity of oleuropein to down regulate the expression of TNF-α, and the consequent inhibition of the expression of the monocyte chemotactic protein-1 and vascular cell adhesion molecule [169].

### 6.4. Metabolic Diseases

Olive oils have demonstrated, in animal studies, a strong capacity to improve blood glucose concentration and diabetic complications at the enzymatic level, their mechanism of action being assigned to their antioxidant activity [170]. Moreover, this fact has been supported by the capacity of bioactive compounds of olive oil to inhibit the gluconeogenesis pathways in liver, as a result of the inhibition of glucose-6-phosphatese enzyme, besides the capacity to induce a significant enhancement in the hepatic catalase activity in vivo [200]. In the same way, Al-Azzawie and Alhamdani [162] demonstrated the capacity of the long-term intake (16 weeks) of oleuropein to restore glucose blood levels in diabetic rabbits [162]. These functions have been also attributed to hydroxytyrosol, while the normoglycemic effect of both oleuropein and hydroxytyrosol has been related to their ability to restrain the oxidative stress, which is responsible for several pathological complications in diabetes [201]. Moreover, some of the benefits observed can be clearly related to synergetic effects between distinct compounds, which is reinforced by the health benefits observed in vivo, including the reduction of oxidative stress and the plasmatic levels of free cholesterol, as well as the inhibition of the atherosclerosis process. These processes were observed in works involving the direct intake of olive oil, either by humans, or using animal models, showing that these effects can be related to a regular intake of EVOO, even though the consumption rates involved were rather high, if compared to a normal Mediterranean diet (Table 5).

### 6.5. Inflammation

Regarding the anti-inflammatory effect of polyphenols of olive oil, in vitro and in vivo studies have allowed to describe this biological activity. Thus, resorting to cell culture assays, addressed to evaluate the role of polyphenols of olive oil regarding inflammation inhibition, it was reported the inhibition of VCAM-1 (Vascular Cell Adhesion Molecule-1) expression [171], whilst other bioactive compounds, namely gallate or vitamin E, reduced the expression of VCAM-1 and ICAM-1 (Intercellular Adhesion Molecule-1) [202,203].

Data published to date have shown a reduction in the inflammatory markers after 2-year of dietary intervention in patients affected by metabolic syndrome [204]. In the same way, Estruch and others [204] reported a reduction of inflammatory markers after 3 months of a Mediterranean diet consumption (including a high proportion of olive oil), based on a randomized study with 772 participants at high risk for cardiovascular disease [205]. This biological activity was further demonstrated by assessing the capacity of virgin olive oil to reduce the systemic levels of the inflammatory markers interleukin-6 and C-reactive protein in comparison with those volunteers consuming refined olive oil [206]. Concerning the expression of adhesion molecules in vivo, changes regarding ICAM in mononuclear cells, associated with a 2-months consumption of monounsaturated fatty acids, have been reported [161].

Oleocanthal, present in EVOO, also exerts anti-inflammatory activity comparable to the non-steroidal compound ibuprofen [139,161], being promoted as a valuable contributor to the reduction of chronic inflammatory response by inhibiting COX-1 and COX-2 enzymes in a dose dependent manner in vivo. By modifying the level of COX enzymes, oleocanthal attenuates the synthesis of prostaglandins E2 [167]. The anti-inflammatory activity of this compound was further supported by diverse in vitro studies that allowed to describe its modulatory effect on the expression of inflammatory mediators (interleukin 6 and macrophage inflammatory protein-1α) and markers (interleukin-1, tumour necrosis factor-α, and granulocyte-macrophage colony stimulating factor) in chondrocytes and macrophages [183]. This achievement reinforces the role of oleocanthal as a critical contributor to the health benefits associated with the Mediterranean diet and, especially, the dietary intake of olive oil, mainly in the ‘extra virgin’ form [196] (Table 4).

## 7. Conclusions

The health benefits recognized to the consumption of olive oil, mainly in the ‘extra virgin’ form, which preserves the constituents responsible for most of its biological activities, are mainly due to their phytochemical contents. Moreover, the factors with visible impact on the metabolism of these compounds in olives are well known, comprising cultivar, agronomical practices, ripening, and biotic and abiotic stresses, causing a remarkable variability, while the oil extraction solely resorting to mechanical processes allows the preservation of these compounds in the final product to be consumed—olive oil.

Among the phytochemical compounds that have been described to present date, secoiridoids, which include oleuropein, and some of its derives, represent the most important group of compounds from olive oil, for which several biological activities have been described, and their mechanisms of action fully understood. According to the data collected, it can be assumed that for some of these bioactive compounds present in olive oil, such as tyrosol, hydroxytyrosol, oleuropein and oleocanthal, any relevant activity corresponding to an IC_50_/EC_50_ around 10 µM, seems to be realistic to occur in a noticeable extent in vivo due to a normal and regular intake of EVOO, if we account with the quantities presented by this foodstuff, and their absorption efficiency.

Furthermore, new analytical methods, and improvement of previous established analytical approaches, will allow a rapid assessment of distinct samples, with enhanced sensitivity, allowing the identification of new compounds, which could be related to some biological activities or health benefits from olive oils, and further assessed. In this sense, while some of the compounds cannot be detected in representative amounts in plasma, after consumption, they have shown enough potential in vitro to be regarded as valuable candidates to be applied as pharmaceuticals, or as parent molecules to be used for the development of newly compounds, resorting to directed synthesis, to target specific biological receptors. In this connection, even though a plethora of biological activities have been described to present date, there is still a lack of comprehension, regarding some of the mechanisms of action, while the description of the bioactive compounds’ structural features, and their relationship with the activity magnitude, would allow the establishment of new SAR’s—leading to a rationale design of newly synthesised compounds with enhanced biological activity.

## Figures and Tables

**Table 1:** Phenolic alcohols and acetate derivatives described in olives and virgin olive oil.

Phenolic Class	Compound	Substituent					Reference
		R_2_	R_3_	R_4_	R_5_	R_6_	
Benzoic acids 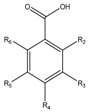	3-Hydroxybenzoic acid	-H	-OH	-H	-H	-H	[81]
	4-Hydroxybenzoic acid	-H	-H	-OH	-H	-H	[85]
	Protocatechuic acid	-H	-OH	-OH	-H	-H	[7,86,87]
	2,4-Dihydroxybenzoic acid	-OH	-H	-OH	-H	-H	[88]
	2,6-Dihydroxybenzoic acid	-OH	-H	-H	-H	-OH	[88]
	Gallic acid	-H	-OH	-OH	-OH	-H	[87]
	Gentisic acid	-OH	-H	-H	-OH	-H	[89]
	Vanillic acid	-H	-OCH_3_	-OH	-H	-H	[89,90,91]
	Syringic acid	-H	-OCH_3_	-OH	-OCH_3_	-H	[92]
Cinnamic acids 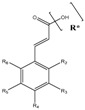	*o*-Coumaric acid	-OH	-H	-H	-H	-H	[87]
	*m*-Coumaric acid	-H	-OH	-H	-H	-H	[93]
	*p*-Coumaric acid	-H	-H	-OH	-H	-H	[85,89]
	Caffeic acid	-H	-OH	-OH	-H	-H	[85,87]
	Hydroxycaffeic acid	-OH	-OH	-OH	-H	-H	[94]
	Ferulic acid	-H	-OCH_3_	-OH	-H	-H	[85,89,90]
	Sinapic acid	-H	-OCH_3_	-OH	-OCH_3_	-H	[8]
	Chlorogenic acid 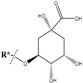	-H	-H	-H	-H	-H	[85]
Hydroxycinnamic acid derivatives	Verbascoside 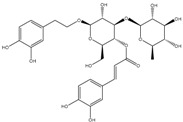	-	-	-	-	-	[95]
Phenylacetic acids 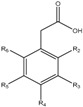	*p*-Hydroxyphenilacetic acid	-H	-H	-OH	-H	-H	[85]
	3,4-Dihydroxyphenylacetic acid	-H	-OH	-OH	-H	-H	[88]
	Homovanillic acid	-H	-OCH_3_	-OH	-H	-H	[85,96]
Other phenolic acid	3-(3,4-Dihydroxyphenyl)propanoic acid 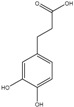	-	-	-	-	-	[92]
Phenolic alcohols 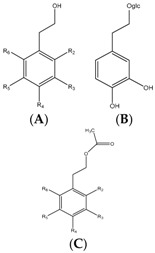	Tyrosol (**A**)	-H	-H	-OH	-H	-H	[91]
	Hydroxytyrosol (**A**)	-H	-OH	-OH	-H	-H	[91]
	3,4-Dihydroxyphenyl)ethanol-glucoside (**B**)	-H	-H	-H	-H	-H	[92]
	Hydroxytyrosol acetate (**C**)	-H	-OH	-OH	-H	-H	[89,91,97]
	Tyrosol acetate (**C**)	-H	-H	-OH	-H	-H	[89,93]

**Table 2:** Secoiridoids, lignans, hydroxy-isochromans and flavonoids described in olives and virgin olive oil.

Phenolic Class	Compound	Substituent						Reference
		R_1_	R_2_	R_3_	R_4_	R_5_	R_6_	
Seicoiridoids	Decarboxymethyloleuropein aglycon	-OH	-	-	-	-	-	[89,112,113]
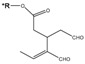	Oleocanthal	-H	-	-	-	-	-	[89]
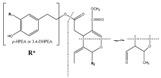	Oleuropein aglycon	-OH	-OH	-	-	-	-	[89,97]
	Ligstroside aglycon	-H	-OH					[90,97]
	Aldehydic form of oleuropein aglycon	-OH	-OH	-	-	-	-	[81]
	Aldehydic form of ligstroside aglycon	-H	-OH	-	-	-	-	[81]
	Oleuropein	-OH	-O-Glc	-	-	-	-	[7,88,96]
Lignans 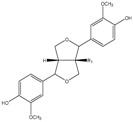	(+)-Pinoresinol	-H	-	-	-	-	-	[93]
	(+)-1-Acetoxypinoresinol	-OCOCH_3_	-	-	-	-	-	[89,97,112]
	(+)-1-Hydroxypinoresinol	-OH	-	-	-	-	-	[92]
Hydroxy-isochromans 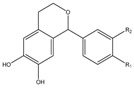	1-Phenyl-6,7-dihydroxyisochroman	-H	-H	-	-	-	-	[88,92]
	1-(3’-Methoxy-4’-hydroxy)phenyl-6,7-dihydroxyisochroman	-OH	-OCH_3_	-	-	-	-	[88,92]
	Luteolin-5-glucoside	-OH	-H	-Glc				
Flavones 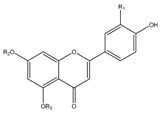	Apigenin	-H	-H	-H	-	-	-	[91,97,114]
	Apigenin-7-glucoside	-H	-Glc	-H				
	Apigenin-7-rutinoside	-H	-Rut	-H				
	Luteolin	-OH	-H	-H	-	-	-	[95,114]
	Luteolin-7-glucoside	-OH	-Glc	-H				
	Luteolin-5-glucoside	-OH	-H	-Glc				
Flavonols 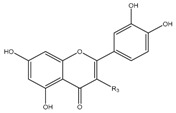	Quercetin	-	-	-OH	-	-	-	[7]
	Quercetin-3-rutinoside	-	-	-Rut	-	-	-	[7,115]
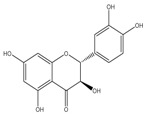	(+)-Taxifolin	-	-	-	-	-	-	[87]
Other phenolic compounds 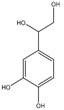	3,4-Dihydroxyphenylglycol	-	-	-		-	-	[97,114]

Rut, rutinoside; Glc, glucoside.

**Table 3:** HPLC procedures in separation of different classes of phenolic compounds.

Stationary Phase	Mobile Phase			*T* (°C)	Flow Rate (mL/min)	*λ* (nm)	Compounds Identified	Reference
	A	B	C					
Luna C18 150 × 2 mm, 5.0 µm	0.1% Formic acid 99.9% Water	95% MeCN 4.9% Water 0.1% Formic acid	-	30	0.40	210–600	Phenolic acids; Phenolic alcohols; Secoiridoids; Lignans; Flavonoids.	[128]
Kinetex C18 100 × 4.6 mm, 2.6 µm	100% Water	100% Acetonitrile	100% Methanol	40	1.25	-	Phenolic acids; Phenolic alcohols; Secoiridoids; Lignans.	[129]
Luna C18 250 × 4.6 mm, 5.0 µm	0.1% Acetic acid 99.9% Water	100% Acetonitrile	-	-	0.60	275	Phenolic acids; Phenolic alcohols; Secoiridoids.	[130]
Zorbax Eclipse Plus RP-C18 150 × 4.6 mm, 1.8 µm	0.25% Acetic acid 99.75% Water	100% Methanol	-	RT ^Z^	0.80	-	Secoiridoids; Lignans; Flavonoids.	[20]
Inertsil ODS-3 250 × 4.6 mm, 5 µm	2% Formic acid 98% Water	100% Methanol	-	22	0.85	240, 280, 320	Phenolic acids; Phenolic alcohols; Flavonoids.	[131]
Zorbax SB-C18 250 × 4.6 mm, 5 µm	5% Acetic acid 95% Water	100% Methanol	100% Acetonitrile	30	1.00	240, 280, 335	Phenolic acids; Phenolic alcohols; Flavonoids.	[30]
Hypersil Gold QRP-18 250 × 4.6 mm, 3 µm	100% Formic acid	100% Acetonitrile	100% Methanol	30	0.80	240, 280, 330	Phenolic acids; Phenolic alcohols; Flavonoids; Secoiridoids.	[132]
Spherisorb S3 ODS2 250 × 4.6 mm, 5 µm	5% Acetic acid 95% Water	100% Methanol	100% Acetonitrile	30	1.00	280	Phenolic acids; Phenolic alcohols; Secoiridoids; Lignans; Flavonoids.	[133]
Zorbax SB-C18 2.1 × 50 mm, 1.8 µm	0.2% Acetic acid 99.8% Water	100% Acetonitrile	-	30	0.40	-	Phenolic acids; Phenolic alcohols; Secoiridoids; Lignans.	[120]
XDB-C18 4.6 × 50 mm, 1.8 µm	0.5% Acetic acid 1% Acetonitrile 98.5% Water	99.5% Acetonitrile 0.5% Acetic acid	-	15	0.60	254, 280, 310, 350	Phenolic acids; Phenolic alcohols; Secoiridoids; Flavonoids	[123]
Zorbax C18 4.6 × 150 mm, 1.8 µm	0.5% Acetic acid 99.5% Water	100% Acetonitrile	-	30	1.50	240, 280	Phenolic alcohols; Secoiridoids; Flavonoids; Lignans.	[54,124]
Hypersil MOS 2.1 × 100 mm, 5 µm	0.5% Acetic acid 1% Acetonitrile 98.5% Water	99.5% Acetonitrile 0.5% Acetic acid	-	25	0.50	255, 260, 275, 280, 310, 320, 325, 340, 350	Phenolic acids; Phenolic alcohols; Flavonoids	[123]

^Z^ Room temperature. Mobile phase: Terms A, B, and C refer to the different phases used during gradient.

**Table 4:** Studied effects of Extra Virgin Olive Oil (EVOO) in vivo.

Compound/Matrix	Activity	Model and Dosis/Intake	Significant Effects	Reference
Hydroxytyrosol	Reduction of oxidative stress	Humans, intake of 50.0 mL of oil with 4.2 mg of hydroxytyrosol and 39.5 mg of oleuropein	~35% reduction in the urinary excretion of 8-iso-PGF2α	[163]
Oleuropein				
	Anti-tumour activity; prevention of skin and breast cancer	Hairless Mice, intake of oleuropein extract (10 mg kg^−1^ of body weight)	Significantly reduced the incidence and growth of tumours	[164]
	Reduction of plasmatic levels of free cholesterol	Rabbits, standard food supplemented with olive oil 10% (*w/w*) and 7 mg kg^−1^ oleuropein	Observed oleuropein aglycone plasmatic levels of 0.892 µM, and LDL increase of 90%	[165]
	Protection against heart damage	Ischemia reperfusion in rats, pre-treatment with 20 µg g^−1^ before ischemia	Significant time-dependent decrease in creatine kinase and reduction of glutathione release	[166]
	Hypoglycemic and antioxidant effect	New Zealand male rabbits, diabetes induced, 20 mg kg^−1^ body weight of oleuropein	Significant decline in plasma and erythrocyte MDA reached at week 10	[162]
Oleanolic acid	Inhibition of cancer proliferation	Mice, HepG2 cells subcutaneously implanted in mice, intraperitoneal injection of OA	75 or 150mg/kg/day led to tumour inhibitory ratios of 31.72% and 57.24%, respectively	[167]
Oleocanthal	Suppression of tumorigenicity	Chicken embryos, implantation of HT29 tumoral cells	~50% reduction of tumour area by treatment with 50 µg/mL	[168]
EVOO	Inhibition of the atherosclerosis process	Humans, daily consumption of 50.0 mL of olive oil	Significant decrease in the inflammatory markers TXB_2_ and LTB_4_	[169]
EVOO	Improve lipid metabolism	Male Wistar rats, cholesterol-free or 1% cholesterol diets, 10 g/100 g EVOO	Reduction of increase in plasma lipids: TC (23.6%), LDL-C (39.3%), TG (19.3%), and TC in liver (36.0%)	[170]
	Increase plasma antioxidant potential		20.6% increase in TRAP, and 23.2% decrease in MDA	

TC—Total Cholesterol; LDL-C—LDL-cholesterol; TG—triglycerides; TRAP—Total radical-trapping antioxidative potential; TC—Plasma total cholesterol; MDA—Malondialdehyde.

**Table 5:** Studied effects of EVOO in vitro.

Compound	Activity	Model	Effective Concentration	Reference
Oleuropein	Scavenge hypochlorous acid in neutrophils	Xantine-xantine oxidase	14.3 µM (EC_50_)	[173]
Hydroxytyrosol			9.1 µM (EC_50_)	
Oleuropein		^1^ PMN ± PMA	29.3 µM (EC_50_)	
Hydroxytyrosol			3.2 µM (EC_50_)	
Oleuropein	Scavenge nitric oxide	Scavenging of nitric oxide generated from 5 mm sodium nitroprusside	~75 µM (EC_50_)	[172]
Caffeic acid				
Hydroxytyrosol				
Oleuropein		*α*1-antiproteinase inactivation assay	(67.2–92.4%) at 1.0 mm	
Caffeic acid				
Hydroxytyrosol				
	Avoiding low-density lipoprotein (LDL) oxidation	Inhibition of LDL copper-induced oxidation	~70% inhibition by 10.0 µM	[159]
Oleuropein	Atheroprotection	Inhibition of VCAM-1	15.0 µM (IC_50_)	[171]
		VCAM-1 mRNA levels	60% reduction by 30 µM	
Hydroxytyrosol			25% reduction by 30 µM	
Erythrodiol	Anti-proliferative	HT-29 (colon adenocarcinoma)	48.8 ± 3.7 µM (EC_50_)	[178]
	Apoptotic activity	HT-29, Caspase-3-like activity	50, 100, and 150 µM—3.2, 4.8 and 5.2 × increase, respectively	
	Anti-proliferative	MCF-7 (human breast cancer cells)	~90% inhibition by 100 µM (24 h)	[179]
Uvaol			~60% inhibition by 100 µM (24 h)	
Oleanolic acid			~85% inhibition by 100 µM (24 h)	
	Inducing apoptotic cell death	HCC (hepatocellular carcinoma) HepG2	Proliferation decrease of 46.5% by 40 μM (24 h)	[167]
Luteolin-7-*O*-glucoside	Anti-proliferative	MCF-7 (human breast cancer cells)	40.85 ± 5.01 µM (IC_50_)	[180]
Oleuropein			12.00 ± 0.62 µM (IC_50_)	
Hydroxytyrosol			24.86 ± 8.15 µM (IC_50_)	
Hydroxytyrosol acetate			28.67 ± 8.10 µM (IC_50_)	
Luteolin-7-*O*-glucoside		T-24 (bladder carcinoma cells)	11.35 ± 0.32 µM (IC_50_)	
Hydroxytyrosol			12.39 ± 2.20 µM (IC_50_)	
Hydroxytyrosol acetate			23.32 ± 0.38 µM (IC_50_)	
Oleuropein			7.59 ± 1.80 µM (IC_50_)	
	Reduction of inflammatory angiogenesis	HUVEC (human vascular endothelial cells), Matrigel assay	Decrease of 40% by 10 µM	[158]
Hydroxytyrosol			Decrease of 55% by 10 µM	
Maslinic acid		HT-29 (colon adenocarcinoma)	28.8 ± 0.9 µg mL^−1^ (EC_50_)	[180]
Squalene	Decrease of radical oxygen species level	MCF10A (breast epithelial cells), H_2_O_2_ assay	~50% reduction by 50 µM (24 h)	[181]
Oleocanthal	Inducing apoptotic cell death	ARH-77 (multiple myeloma cell line)	Proliferation decrease of ~80% by 50 μM (24 h)	[182]
	Inhibition of carcinogenesis	JB6 Cl41 (cell line sensitive to carcinogenesis)	3 µM inhibits the expression of p-ERK 1/2, and P-p90rsk	[168]
	Inducing apoptotic cell death	SK-BR-3 (Human breast cancer cell line)	Proliferation decrease of ~10% by 15 µM	
		HCT-116 (Human colon colorectal carcinoma)	Proliferation decrease of ~70% by 15 µM	
	Inhibition of metastatic process	MCF7 (nonmetastatic human breast cancer)	18 µM (IC_50_)	[174]
		MDA-MB-231(highly metastatic human breast cancer)	15 µM (IC_50_)	
		PC-3 (human prostate cancer)	20 µM (IC_50_)	
	Anti-inflammatory activity	Inhibition of MIP-1α in J774 macrophages	~50% inhibition by 50 µM (24 h)	[183]
		Inhibition of IL-6 in J774 macrophages	~60% inhibition by 50 µM (24 h)	
		Inhibition of MIP-1α in ATDC5 chondrocytes	~90% inhibition by 50 µM (24 h)	
		Inhibition of IL-6 in ATDC5 chondrocytes	~75% inhibition by 50 µM (24 h)	
Oleocanthal (−)	Anti-inflammatory activity	COX1 inhibition	56.1 ± 3.2% inhibition by 25 µM	[161]
Oleocanthal (+)			68.0 ± 15.2% inhibition by 25 µM	
Oleocanthal (−)		COX2 inhibition	56.6 ± 9.5% inhibition by 25 µM	
Oleocanthal (+)			41. 3± 15.9% inhibition by 25 µM	

^1^ PMN, human polymorphonuclear neutrophils; PMA, phorbol-12-myristate-13 acetate.
